# CaMKIIδ gene editing- A base hit for the heart

**DOI:** 10.20517/jca.2023.11

**Published:** 2023-03-22

**Authors:** Christopher J. Walkey, William R. Lagor

**Affiliations:** Department of Integrative Physiology, Baylor College of Medicine, Houston, TX 77030, USA.

**Keywords:** Base editing, heart, ischemia-reperfusion, aav, crispr

In the January 13, 2023 issue of *Science*, Lebek and colleagues demonstrate the potential broad utility of *in vivo* base editing as a gene therapy for heart disease^[1]^. Following myocardial infarction, it is a race against time to begin thrombolytic therapy and percutaneous coronary intervention to restore blood flow to the infarcted region. Revascularization of the infarcted artery is performed to prevent cardiomyocyte death, fibrosis, and heart failure. However, these procedures come with their own adverse sequelae of ischemia-reperfusion injuries, which include myocardial stunning, microvascular obstruction, arrhythmias, and lethal injury^[2]^. Therapies that can either prevent cardiomyocyte injury and death, or promote regeneration of the infarcted tissue are desperately needed. Such approaches should use common molecular pathways that are applicable to all patients.

Calcium (Ca^2+^) plays an essential role in excitation-contraction coupling in the heart and is also an important signaling molecule that controls metabolism. Ca^2+^/calmodulin-dependent protein kinase IId (CaMKIIδ) is a critical regulator of cell signaling in cardiomyocytes, but its hyperactivation is directly implicated in myocardial infarction^[3]^, ischemia/reperfusion injury^[4]^, arrhythmias^[5]^ and heart failure^[6]^. Hyperactivation occurs through the oxidation of two methionine residues, Met281 and Met 282, which prevents the interaction between CaMKIIδ’s catalytic domain and an autoinhibitory domain. Genetically engineered knock-in mice with oxidation-resistant mutations of these residues are protected from atrial fibrillation^[7]^ and mortality after heart failure^[8]^.

The authors sought to install substitutions of valine for these methionines to render the CaMKIIδ protein resistant to oxidation and hyperactivation in a targeted, on-demand manner. This creative strategy disrupts a pathological signaling pathway, where a modest change would be made to the CaMKIIδ protein, thereby maintaining its expression. To achieve such precise edits, the authors tested adenine base editors for their ability to convert the ATG Met codon to GTG. The Adenine Base Editors are engineered versions of the Cas9 nuclease that can bind DNA, but not fully cut^[9]^. Instead, a tethered deaminase domain promotes the conversion of A:T to G:C dinucleotides within a small “editing window”. A major advantage of this technology is the ability to install precise single nucleotide changes without double-strand breaks^[10]^. Application of base editors requires rigorous testing to avoid unintended modifications adjacent to the target site (bystander edits) as well as elsewhere in the genome (off-target edits).

Two different base editors and multiple guide RNAs (gRNA) were screened for their ability to edit the human CaMKIIδ sequence. The most efficient gRNA could install both of the required M281V and M282V substitutions with high efficiency, although it also produced a bystander edit at a neighboring residue resulting in an H283R substitution. This base editor and gRNA were further evaluated in human induced pluripotent stem cells, where they produced a remarkable frequency of 75% homozygously edited clones. Human iPSCs were also used to test specificity against predicted sites in the human genome. This analysis was particularly important in this case, given the multiple CaMKII homologs present in the genome. Off-target editing at the highly similar CamKIIβ gene was found at a level equivalent to that of CamKIIδ, but this homolog is not expressed in cardiomyocytes. Another off-target site was also found in an intronic region of *DAZL* gene, which was predicted to be inconsequential. Balancing efficiency and specificity is a challenge, especially for base editing, and the authors did a great job identifying the best components. Edited iPSC clones were then differentiated into cardiomyocytes to probe the functional consequences on CaMKIIδ activity. Using an *in vitro* model of ischemia reperfusion injury with a hypoxia chamber, it was clear that the edited cells expressed normal CaMKIIδ levels but without methionine oxidation. As a consequence, CaMKIIδ autophosphorylation and catalytic activity were reduced. Downstream RyR2 phosphorylation was reduced, and Ca^2+^ handling disturbances that are predictive of arrhythmias were inhibited.

To test the therapeutic potential of CaMKIIδ base editing, the authors delivered the base editing machinery using two adeno-associated viral vectors (AAV9) employing a split-intein strategy. Adult mice underwent ischemia-reperfusion (IR) injury and received the AAV9 vectors directly at the injured region. Incredibly, four weeks later, the cardiac function of the edited mice was restored to the levels of sham controls. This was evident by improvements in fractional shortening, reduced left ventricular end-diastolic diameter, and higher ejection fraction. Efficient genome editing was achieved in the heart, in the range of 46% of CaMKIIδ transcripts. Consistent with strong protection from the initial cardiac injury, CaMKIIδ-edited hearts had fewer apoptotic cells, less inflammatory cell infiltration, and decreased fibrotic scarring. RNA-Seq provided global evidence of restored cardiac function in the edited mice post-treatment.

Rigorous optimization and testing of the gRNA was performed. Highly efficient editing was achieved both in human cells as well as in a mouse disease model. Although there was an assessment of off-target editing, further work is required for clinical translation. The most pressing question would be the functional consequences of the bystander H283R edit in CaMKIIδ. Would this have an adverse effect on the structure or activity of this important kinase? What about editing closely related family members like CaMKIIβ? Is it not a concern if expressed only in other tissues, even if the genome editing machinery is delivered directly to the heart? Also, does ablating the ability of CaMKIIδ to hyperactivate in response to oxidative stress have consequences independent of IR injury? After all, the affected methionines have been preserved through evolution. In addition to gRNA-dependent off-target editing, base editors can also have nonspecific off-target activity resulting from transient interactions between the deaminase and DNA or RNA. While these are believed to be largely random and less frequent with ABEs versus cytosine base editors, they remain a concern.

Dual AAV9 delivery of the split-intein base editor system, necessitated by the limited packaging capacity of AAVs, was remarkably effective. This was accomplished with local injection of the heart at the site of injury. Local injection promotes co-delivery of both vectors to the same cardiomyocytes. Systemic administration of very high dose AAV vectors (>1 × 10^14^ vector genomes/kg) for neuromuscular gene therapies has recently resulted in multiple patient deaths due to hepatotoxicity and/or thrombotic microangiopathy (TMA). In this context, therapies that directly target an infarcted region of the heart at substantially lower doses of AAV are particularly appealing.

Ensuring safety will also require consideration of immune responses to AAV vectors as well as the base editor itself. Neutralizing antibodies to the AAV capsid is generally an exclusion criterion for human gene therapy trials and, in many cases, can eliminate 50% of the patient pool. In a similar way, a large fraction of the population also has pre-existing immunity to the *Streptococcus pyogenes* Cas9 protein, which is the core of this adenine base editor. Recent work in dog models of Duchenne muscular dystrophy have shown that cytotoxic T-cells can infiltrate and destroy edited myofibers expressing CRISPR/Cas9^[11]^. However, lifesaving new therapies must carefully weigh the risks and potential benefits. For myocardial infarction, IR, and heart failure, the benefit is huge. It is possible that a self-deleting Base Editing system could be employed to improve the safety margin of such a vector. In the future, it may even be possible to transiently deliver the BE system as mRNA or ribonucleoprotein directly to cardiomyocytes, avoiding this problem altogether.

Several aspects of this paper are particularly appealing with respect to translation. One is that gene therapy could be applied locally during coronary angiography to revascularize the infarcted artery. However, it may be necessary to inject the AAV9 directly into the myocardium to ensure efficient transduction. This could be tested in pigs or a larger animal model. If it works well, this could be an “off-the-shelf” therapy that could be very broadly applied. One question that remains is the window for intervention. In the mouse model, AAV were delivered exactly at the time of injury. Would it still work in humans if there was a delay in the order of hours to days? Also, how much of the beneficial effect reported here is injury prevention versus correction? Combining this approach with strategies that temporarily promote cardiomyocyte regeneration may add value. Lastly, there are logistical challenges associated with such a clinical trial. A major advantage is the potentially large pool of participants for trials, who share a common and unfortunate human life event, rather than a rare genetic disease. Conversely, survival is a high bar for success in a mixed patient population, so surrogate readouts of efficacy will be important to consider.

This work is conceptually exciting as one of the first base editing preclinical studies for cardiac disease. It demonstrates that highly efficient editing can be achieved *in vivo*. Furthermore, it uses an elegant approach to modify a pathogenic pathway for a favorable outcome. It provides proof of principle that base editing could also be applied to common life-threatening conditions. Given the recent approvals of AAV gene therapies for congenital blindness, spinal muscular atrophy, and Hemophilia B, it seems the cardiac gene therapy field is long overdue for its own success story.

## Data Availability

Not applicable.
